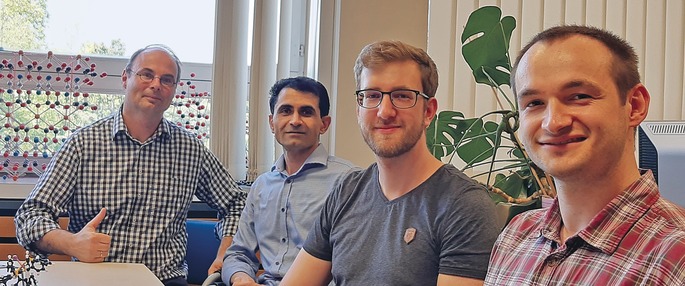# Regioselective Functionalization of [2.2]Paracyclophanes: Recent Synthetic Progress and Perspectives

**DOI:** 10.1002/anie.201904863

**Published:** 2019-10-30

**Authors:** Zahid Hassan, Eduard Spuling, Daniel M. Knoll, Stefan Bräse

**Affiliations:** ^1^ Institute of Organic Chemistry (IOC) Fritz-Haber-Weg 6 76131 Karlsruhe Germany; ^2^ 3DMM2O—Cluster of Excellence Institute of Organic Chemistry (IOC) Karlsruhe Institute of Technology (KIT) Germany; ^3^ Institute of Toxicology and Genetics (ITG) Karlsruhe Institute of Technology (KIT) Hermann-von-Helmholtz-Platz 1 76344 Eggenstein-Leopoldshafen Germany

**Keywords:** [2.2]paracyclophanes, chirality, functional parylenes, π-stacked polymers, regioselective synthesis

## Abstract

[2.2]Paracyclophane (PCP) is a prevalent scaffold that is widely utilized in asymmetric synthesis, π‐stacked polymers, energy materials, and functional parylene coatings that finds broad applications in bio‐ and materials science. In the last few years, [2.2]paracyclophane chemistry has progressed tremendously, enabling the fine‐tuning of its structural and functional properties. This Minireview highlights the most important recent synthetic developments in the selective functionalization of PCP that govern distinct features of planar chirality as well as chiroptical and optoelectronic properties. Special focus is given to the function‐inspired design of [2.2]paracyclophane‐based π‐stacked conjugated materials by transition‐metal‐catalyzed cross‐coupling reactions. Current synthetic challenges, limitations, as well as future research directions and new avenues for advancing cyclophane chemistry are also summarized.

## Graphical abstract

**From synthesis to function**: [2.2]Paracyclophane (PCP) is a prevalent scaffold in asymmetric synthesis, π‐stacked polymers, energy materials, and functional parylene coatings that finds broad applications in bio‐ and materials science. This Minireview highlights the enormous progress made in PCP chemistry that has enabled the fine‐tuning of its structural and functional properties.

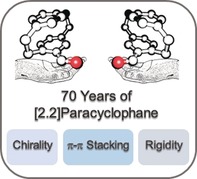

## 1 Introduction and History

### 1.1 PCP: From Synthetic Curiosity to Inspiring Functions

**Figure 1:**
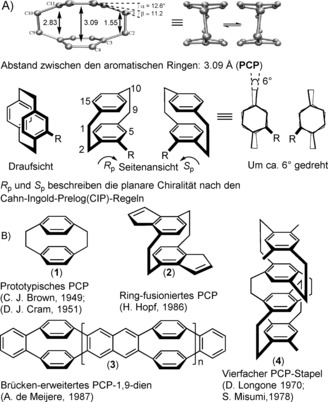
A) Structural parameters in Ångstrom [Å] determined by X‐ray crystallography. B) Representative PCP‐based ring‐fused, ring‐extended, and quadruple π‐stacked systems.

[2.2]Paracyclophane was discovered by Brown and Farthing in 1949 by the gas‐phase pyrolysis of *para*‐xylene under low pressure.^1^ Two years later, Cram and Steinberg reported the first synthesis of this novel and intriguing compound by intramolecular cyclization.^2^ Since then, this rather unusual “bent and battered”^3^ strained organic scaffold has sparked numerous investigations and received attention because of its intriguing structural and electronic properties. These result in unique physical and chemical behavior, accompanied by aesthetically pleasing structures.^4^ [2.2]Paracyclophane (**1**) consists of two cofacially stacked, strongly interacting benzene rings (decks) with an average ring‐to‐ring distance of 3.09 Å. This is far less than the standard van der Waals distance of 3.40 Å observed between layers in graphite.^5^ The phenyl rings in PCP are stacked cofacially in proximity, held together by two ethylene “bridges” (2.83 Å) at the bridgehead carbon atoms in a *para* orientation (see molecular structure in Figure 1, top).^6^ The stacking of the two benzene rings leads to a strain energy of about 31 kcal per mole for PCP,^7^ and causes a distortion so that the two benzene rings are forced to bend out from planarity at the bridgehead carbon atom by 12.6° out of the benzene plane. This provides the basis for distortion abnormalities from aromatic planarity and for correlation between the properties and unusual reactivity behavior. In larger [*n.n*]cyclophanes, such as [3.3]paracyclophanes and [4.4]paracyclophanes,^8^ the longer propyl or butyl bridges between the two benzene rings allow the decks to be further apart (3.3 Å avg.) and, therefore, less strained (ca. 12 kcal mol^−1^), while [6.6]paracyclophane is nearly strain‐free (2 kcal mol^−1^), comparable to an open‐chain compound.^3^ The strain energy, conformations, and rotational barriers of the [2.2]‐, [3.3]‐, and [4.4]paracyclophanes predicted by DFT studies are in close agreement with experiment.^9^

The pioneering research on [2.2]paracyclophane chemistry has been predominantly mechanistic in nature, demonstrating structure/reactivity relationships, and understanding the unusual physical/chemical properties of PCP. In particular, the development of synthetic methods reported by the groups of Hopf,^10^ de Meijere,^11^ Longone,12a Misumi,12b and others have greatly actuated the formation of various molecular stacks such as ring‐fused (**2**), bridge‐extended (**3**), and quadruple stacks (**4**) as well as other chemical topologies to examine molecular strain and transannular π‐π interactions.^13^

[2.2]Paracyclophane chemistry has evolved from functional molecules to functional materials and from synthetic curiosity to emerging applications in asymmetric synthesis, energy materials, π‐stacked polymers, and functional parylene coatings (polymer made by polymerization of PCP induced by vapor‐phase pyrolysis).^14^ Numerous material applications dealing with planar chirality and through‐space conjugation have been the subject of excellent reviews.^15^ With such a large body of work, it is impossible to cover every aspect here. The aim of this Minireview is to describe most of the recent advances and some landmark results that are particularly appealing for chemists, material scientists, and engineers aiming to work in the areas of 1) cyclophane chemistry, 2) design and development of new ligand and catalyst systems, 3) asymmetric synthesis, and 4) advanced polymer materials.

### 1.2 Structure–Property Relationships of PCPs: From Asymmetric Synthesis to Materials Applications

**Figure 2:**
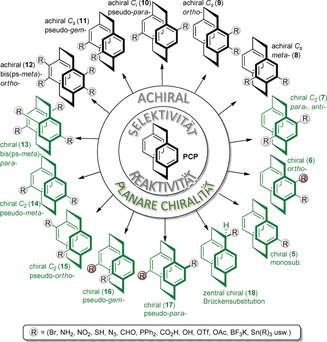
Common substitution patterns of mono‐ and disubstituted PCP with stereochemical description; ps=pseudo. The top view of PCP is given for clarity.

Using carefully chosen reaction parameters and transformation steps, the PCP core allows different substituents to be positioned regioselectively at either only one (Figure 2; **5**–**9**) or both benzene rings (Figure 2; **10**–**17**). Functional moieties can be incorporated directly onto the benzene rings (such monosubstituted PCPs possess planar chirality) or the ethylene bridges, such as in PCP **18**, which lead to centrally chiral compounds. The substitution pattern of PCPs heavily influences the nature of the compound and even minor changes can alter its properties significantly.

**Figure 3:**
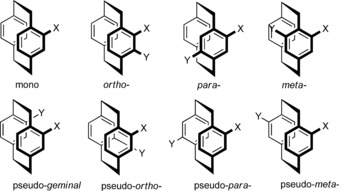
Aromatic substitution pattern of disubstituted [2.2]paracyclophane with the relative nomenclature.

Particular descriptors are employed to designate the relationship of the two substituents in disubstituted PCP derivatives. For disubstitution on one phenyl ring, the conventional prefixes of *ortho‐*, *para‐,* and *meta‐* are used, while disubstitution on both benzene rings gives rise to pseudo‐*geminal*, pseudo‐*meta*, pseudo‐*para*, and pseudo‐ *ortho* prefixes (Figure 3).

Appropriately functionalized derivatives of PCP have found ample applications as one of the most successful, versatile, and commonly used classes of planar chiral ligands or chiral catalysts for stereoselective syntheses, second only to ferrocene‐based systems (e.g. the JosiPhos family). The application of PCP as a planar chiral ligand has been extensively investigated by the groups of Rossen and Pye,^16^ Rozenberg,^17^ Rowlands,^18^ Bolm,^19^ Paradies,^20^ and Bräse.^21^

**Figure 4:**
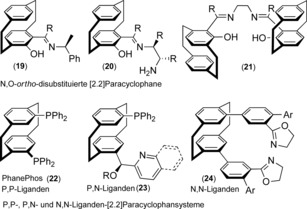
Representative chiral [2.2]paracyclophane‐based N,O, P,N‐, N,N‐, and P,P‐ligands used in asymmetric catalysis.

Our research groups have devoted significant efforts over the past two decades to the development of new and generally useful classes of enantiomerically pure mono‐ and disubstituted [2.2]paracyclophane‐based planar chiral ligands and catalysts. They have been successfully employed for various synthetically important stereocontrolled and enantioselective transformations, for example, the addition of alkyl, aryl, alkynyl, and alkenyl zinc reagents to aromatic and aliphatic aldehydes and imines.^21^ In this Minireview, we confine our discussion only to the molecular design and specific structural features of some chiral representatives, based on the nature of the chirality, donor atoms, and their denticity, such as di‐, tri‐, and tetradentate N,O‐[2.2]paracyclophane ligands (**19**–**21**). Some prominent examples of planar chiral PCP ligands are the P,P‐ligand PhanePhos (**22**), mixed P,N‐ligands containing pyridine or quinoline (**23**), as well as N,N‐ligands such as bisoxazoline (**24**), all of which have shown remarkable performance in asymmetric catalysis (Figure 4). A detailed description of their preparations and diverse applications as ligands or catalysts in a wide range of asymmetric syntheses can be found in the literature.^22^

**Figure 5:**
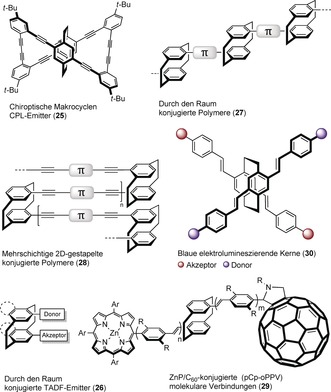
Design of various PCP‐based π‐stacked systems.

The function‐inspired design of through‐space‐conjugated molecular assemblies based on [2.2]paracyclophane and their numerous applications has been a prominent objective in diverse areas of materials research. To engineer discrete π‐stacks of aromatic assemblies, the groups of Chujo,^23^ Bazan,^24^ Collard,^25^ and others^26^ have employed specific PCP precursors with the aim of tuning the photophysical, optoelectronic, and electrochemical features of substantial chemical and industrial importance (Figure 5). Rigidity, stability, planarity, tolerance to moisture, and π‐stacking of the PCP core fulfill the critical requirements for organic electronic materials.

Additionally, remarkable progress has been achieved in the field of cyclophane‐based chiroptical circularly polarized luminescent (CPL) emitters (**25**)^27^ and thermally activated delayed fluorescence (TADF) emitters (**26**),^28^ through‐space‐conjugated π‐stacked polymers (**27**),^29^ multilayered 2D‐stacked materials (**28**),^30^ paracyclophane‐derived molecular junctions in oligo(phenylenevinylene)s (OPVs, **29**),^31^ and basic understanding of interchromophore delocalization in electroluminescent device fabrication (**30**),^32^ to name but a few.

## 2 Selective Mono‐, Di‐, and Multisubstitution of PCP and Molecular Reactivities

### 2.1 Monofunctionalization of [2.2]Paracyclophane

**Scheme 1:**
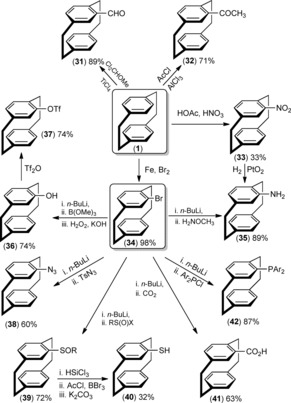
Monofunctionalization of [2.2]paracyclophane.

[2.2]Paracyclophane is chemically stable towards light, oxidation, acids, bases, and temperature up to 180 °C, but it is also often resistant to conventional chemical transformations and, therefore, causing unique synthetic challenges.^33^ PCPs exhibit unusual chemical reactivity because of their remarkable transannular interactions. Additionally, PCP can undergo thermal isomerization at elevated temperatures, consequently changing its electronic, physical/chemical, and spectroscopic properties.^34^ Monofunctionalization of PCP can be achieved by electrophilic aromatic substitution^35^ to install functional groups either in a single step (e.g. CHO (**31**), COCH_3_ (**32**), NO_2_ (**33**), and Br (**34**))^36^ or by quenching the lithio derivative prepared from bromide **34** with various electrophiles, thereby leading to the incorporation of other important moieties such as NH_2_ (**35**), OH (**36**), N_3_ (**38**),^37^ SOR (**39**), SH (**40**),^38^ CO_2_H (**41**), and PAr_2_ (**42**).^39^ These functional groups make PCP an attractive synthon. Functional groups such as the phenol derivatives **36** can be further transformed into triflate (OTf, **37**), toslyate (OTs), and acylation products.^40^ A selection of well‐established synthetic routes for the monofunctionalization of PCP derivatives with different substituents is shown in Scheme 1.

### 2.2 Difunctionalization of PCP at One or Both Benzene Rings: Reactivity/Selectivity

Disubstituted PCPs are accessible starting from monosubstituted PCP. The mechanisms of the regioselective electrophilic aromatic substitution reactions of PCPs and their interconversions were investigated by Cram and Reich.^3^ A strong directing effect by the first substituent on PCP derivatives was observed, which is known as the transannular directive effect.^39^

**Scheme 2:**
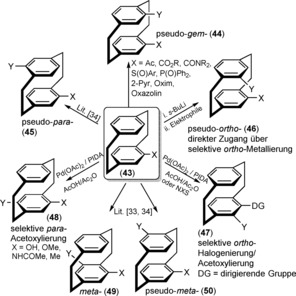
Difunctionalization of [2.2]paracyclophane derivatives. DG=directing group, PIDA=phenyliododiacetate.

The pseudo‐*geminal* regioisomers are more easily accessible and can be efficiently prepared selectively through the electrophilic aromatic substitution of monosubstituted [2.2]paracyclophane derivatives. In the iron‐catalyzed bromination of **1**, modest regioselectivity is observed, with pseudo‐*para*‐ (26 %) and pseudo‐*ortho*‐dibromides (16 %) as the major isolated products accompanied by lesser amounts of the pseudo‐*meta*‐ (6 %) and *para*‐dibromides (5 %).^33^, ^34^ From a geometric point of view, the two appending moieties on the [2.2]paracyclophanes can be held parallel to each other, that is, pseudo‐*geminal* (**44**), antiparallel in pseudo‐*para* (**45**) and *para* arrangements (**48**), and finally two variants of V‐shaped geometries are possible in pseudo‐*ortho* (**46**) and *ortho* (**47**; 60°), *meta* (**49**), and pseudo‐*meta* arrangements (**50**; 120°) as depicted in Scheme 2. The transannular‐directed regioselective bromination to pseudo‐*geminal* PCP derivatives was first reported by Reich and Cram for various carbonyl‐substituted PCP derivatives (**44**), such as carboxylic acid, methyl ester, acetyl derivatives, and nitro‐substituted derivatives.^34^ By employing a thermal isomerization procedure, pseudo‐*meta* isomers (**50**) can be accessed from the readily available *pseudo*‐*geminal* isomer (**44**). The proximity of the two substituents within the pseudo‐*geminal* substitution pattern of isomer **44** drives the equilibrium towards the thermodynamically more favored *pseudo*‐*meta* isomers (**50**) as a result of steric repulsion.^34^ Thus, a broad set of architectural building blocks with different geometrical arrangements, within one plane or with an offset of one plane, can be obtained.

The thermal isomerization proceeds by homolytic cleavage of a two‐carbon bridge to form a diradical, rotation, and subsequent ring closure. The isomerization of pseudo‐*para* products into their isomeric pseudo‐*ortho* derivatives was investigated in detail by the groups of Rozenberg^46^ and Hopf.^41^ Braddock et al. developed cleaner reaction conditions (180 °C, 6 min, in DMF) for a high‐yielding synthesis of up to 38 % for the pseudo‐*ortho*‐dihydroxy[2.2]paracylophane, with simple separation of the two enantiomers by precipitation.^42^ A microwave‐assisted method for the isomerization of pseudo‐*para*‐dibromo[2.2]paracyclophane into the corresponding pseudo‐*ortho*‐dibromo[2.2]paracyclophane in DMF has also proven useful.^43^ Recently, regioselective direct pseudo‐*ortho*‐metalation, *ortho*‐halogenation, and *para*‐selective acetylation through nonconventional functionalization strategies involving activation of carbon–hydrogen bonds have been reported. This is discussed in the upcoming section on nonconventional functionalization strategies for PCP.

### 2.3 Multiple Functionalization of [2.2]Paracyclophane: Regioselective Substitution (Pattern Control)

**Scheme 3:**
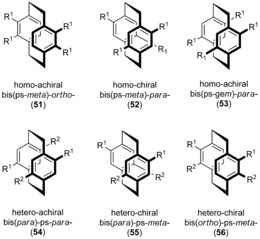
Aromatic substitution pattern of symmetrically tetrasubstituted homo‐ and bis‐bifunctional PCPs with the relative nomenclature.

[2.2]Paracyclophanes with halide and pseudohalide substituents represent versatile modular molecular building blocks that have been incorporated in numerous functional materials.^44^ Tetrasubstituted PCP, one of the most thoroughly investigated precursors, can be accessed by multiple electrophilic substitution reactions (Scheme 3). A regioselective double electrophilic substitution of various disubstituted [2.2]paracyclophanes to obtain symmetrically tetrasubstituted (**51**–**53**) and regioisomeric bis‐bifunctional PCP derivatives (**54**–**56**) has been investigated.^45^ For example, the diacylation of differently substituted diphanols, such as pseudo‐*meta*‐dihydroxy[2.2]paracyclophane, pseudo‐*meta*‐dimethoxy[2.2]paracyclophane, pseudo‐*meta*‐bis(methoxycarbonyl)[2.2]paracyclophane, and bis(methoxycarbonyl) derivatives, constitutes a general and useful approach to obtain several different types of hetero‐substituted, regioisomeric chiral *C*_2_‐symmetric bis‐bifunctional [2.2]paracyclophane derivatives (**54**–**56**). The nomenclature used for homo‐substituted and hetero‐substituted isomers is based on suggestions of Hopf and co‐workers, where the prefix ps represents pseudo.^46^

**Scheme 4:**
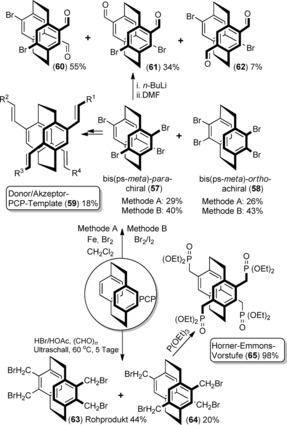
Synthesis of symmetrically tetrasubstituted homo‐ and bis‐bifunctional PCPs.

The use of excess bromine in the presence of an iron catalyst (Scheme 4, method A) leads to two main regioisomeric tetrasubstituted [2.2]paracyclophane products (**57**, **58**) in modest yields of 29 % and 26 %.^39^ Slight modification of the reaction conditions, by leaving [2.2]paracyclophane in liquid bromine in the presence of traces of iodine (Scheme 4, method B), results in the yields of the symmetrically tetrasubstituted bromo[2.2]paracyclophanes **57** and **58** being improved considerably to 40 % and 43 %, respectively.^45^

The palladium‐mediated fourfold cross‐coupling reaction of 4,7,12,15‐tetrabromo[2.2]paracyclophane (**57**) with phenylacetylene, styrenes, or methyl acrylate as well as the nickel‐catalyzed coupling of phenylmagnesium bromide have been investigated.^45^ An alternative route involving the site‐specific coupling of PCP derivatives (**60**–**62**), obtained by selective lithiation followed by quenching with DMF, is often used for the formation of various donor/acceptor phenylenevinylene templates (**59**), which is an ideal core to study the impact of substitution patterns on through‐space charge transfer.^47^ Similarly, bromomethylation of [2.2]paracyclophane through sonication results in the tetrasubstituted tetra(bromomethyl) PCPs bis‐(pseudo‐*meta*)‐*para*‐**63** and bis‐(pseudo‐*meta*)‐*ortho*‐**64**, a precursor to 4,7,12,15‐tetra‐diethylphosphonatemethyl‐substituted [2.2]paracyclophane **65**, known as a Horner–Emmons precursor. This intriguing intermediate gives access to different combinations of styrylbenzene chromophores containing donor and acceptor groups across a [2.2]paracyclophane bridge.^48^

[2.2]Paracyclophane scaffolds can also be prepared using 2,11‐dithia[3.3]paracyclophane intermediates bearing the desired substituents on the aromatic rings in the appropriate configuration, followed by desulfurization.^49^ This route has the advantage that heterocycles can be incorporated into the skeleton. The Diels–Alder cycloaddition of 1,2,4,5‐hexatetraenes with symmetrically or unsymmetrically substituted acetylene to yield the corresponding tetrasubstituted [2.2]paracyclophane derivatives has also been reported.^50^

### 2.4 Conventional versus Nonconventional Functionalization Strategies of [2.2]Paracyclophane

**Scheme 5:**
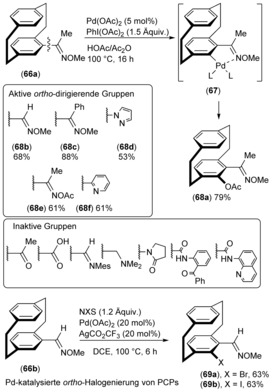
Overview of the performance of the directing groups in the Pd‐catalyzed *ortho*‐acetoxylation of [2.2]paracyclophanes.

Synthetic strategies for the functionalization of a PCP molecule mostly rely on electrophilic substitution reactions or certain transformations via preinstalled functional groups. Despite the significant advances in metal‐catalyzed C−H bond functionalization, the direct functionalization of [2.2]paracyclophane has remained largely unexplored. The nonconventional functionalization approach is atom‐economic with a minimal number of steps, skips prefunctionalization, and can, therefore, minimize tedious synthetic efforts. As a consequence of the chemically similar nature of the C−H bonds in PCP molecules, site selectivity is one of the main challenges in direct functionalization. The first palladium‐catalyzed direct C−H bond acetoxylation of [2.2]paracyclophanes **66** was reported by Bolm and co‐workers, and was suitable for obtaining various *ortho*‐substituted hydroxy[2.2]paracyclophane derivatives **68** via cyclophane‐based palladacycles **67** using 1–5 mol % palladium(II) acetate in combination with iodobenzene diacetate as an oxidant.^51^ The direct *ortho*‐selective acetoxylation can be effectively directed by aldoxime ethers, ketoxime ethers, and esters, with 2‐pyridyl and pyrazole acting as active directing groups on the [2.2]paracyclophane (Scheme 5, top). Although oximes proved to be excellent *ortho*‐directing groups for Pd‐catalyzed *ortho*‐C−H activation, numerous other common directing groups were inactive, either because they were incompatible under the reaction conditions or too bulky (Scheme 5).

Following the pioneering studies on the Pd‐catalyzed *ortho*‐acetoxylation of PCP, a Pd‐catalyzed selective *ortho*‐bromination/iodination procedure employing [2.2]paracyclophane derivative **66 b** was developed. This method provided swifter access to 4,5‐disubstituted [2.2]paracyclophanes via *ortho*‐functionalized intermediates **69 a,b**. However, a 20 mol % catalyst loading is required (Scheme 5, bottom).^52^ The described *ortho*‐selectivity of the C−H bond functionalization in a PCP backbone was achieved with an *O*‐methyloxime directing group. The synthetic value of this procedure was further demonstrated by exemplary conversions of the carbaldehyde and halogen groups.

**Scheme 6:**
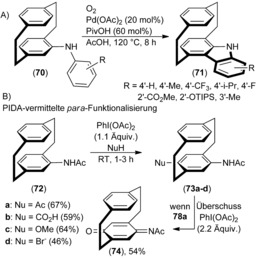
A) Oxidative cyclization. B) PIDA‐mediated *para*‐C−H functionalization of [2.2]paracyclophane, and oxidation to benzoquinone. Nu=nucleophile, PivOH=pivalinic acid, TIPS=triisopropylsilyl.

Subsequently, Bolm and co‐workers extended the scope of the regioselective *ortho*‐C−H functionalization to acidic conditions to synthesize a planar chiral (1,4)carbazolophane **71** by oxidative cyclization under aerated conditions starting from *N*‐phenylamino[2.2]paracyclophane **70** obtained by Hartwig–Buchwald C−N cross‐coupling of the respective anilines and 4‐bromo[2.2]paracyclophane (Scheme 6 A).^53^ In contrast to the *ortho*‐acetoxylation to form **68**, a significantly increased catalyst loading of 20 mol % was used to obtain **71** in 62 % yield. It is noteworthy that the oxidative cyclization does not occur with the N‐methylated derivative. Although the reaction conditions are rather harsh, this procedure is reported to be successful for a wide range of electron‐rich and ‐poor aniline derivatives, which give access to the very interesting class of carbazolophanes. They have the potential to replace the ubiquitously used carbazole group in a number of material science applications, where the increased steric bulk or planar chirality are of key interest.28a

**Scheme 7:**
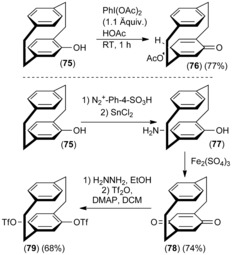
Oxidative *para*‐C−H functionalization of PCP with a quinone intermediate. DCM=dichloromethane, DMAP=dimethylaminopyridine, OTf=triflate.

Recently, the *para*‐C−H functionalization of phenylamino‐ and acetamido‐substituted PCPs (**72 a**–**d**) mediated by phenyliodide diacetate (PIDA) as an oxidant (Scheme 6 B) was reported.^54^ Various nucleophiles such as acetate, formate, methanolate, ethanolate, and bromide could be successfully positioned at the *para*‐position (**73 a**–**d**). Insight into the mechanism was gained when an excess of PIDA was added, which give a benzoquinimine **74** in 54 % yield. The presence of an oxidized ketone intermediate is strongly supported. In a similar manner, benzoquinone **78** was already reported by Cram and Day in 1966 and is a convenient intermediate,^55^ as both the enantiopure precursors **75** are readily available and the benzoquinone **78** can be easily converted into the *para*‐bistriflate **79**, an important synthon in cross‐coupling reactions (Scheme 7).^56^ Efforts in C−H functionalization have been successful because directing groups on PCP change the reactivity of the nearby C−H bond. However, new methods in C−H functionalization (without directing groups) to install active functionalities sequentially either at one or both benzene rings of the [2.2]paracyclophane backbone were only discovered recently by Yu and co‐workers.^57^

## 3 Convenient Synthetic Approaches and Resolution of Key Mono‐, Di‐, and Tetrasubstituted PCPs

The separation of planar chiral PCP derivatives can be a tedious endeavor, especially when larger quantities are needed. To circumvent expensive chiral (semi)preparative HPLC techniques, chiral resolution techniques with derivatizing agents have been reported.^58^, ^59^, ^60^ Various procedures for chiral resolution using optimum chiral auxiliaries, for example, derivatives of l‐amino acids, (+)‐naproxen, (*S*)‐(−)‐camphanoyl chloride, (*S*)‐(+)‐10‐camphorsulfonic acid, and (−)‐menthol, are reported to afford enantiomerically pure mono‐, di, and tetrasubstituted PCP derivatives.^35^, ^61^ In this section, a selection of the most commonly employed methods for the resolution of several key [2.2]paracyclophane derivatives is described.

**Scheme 8:**
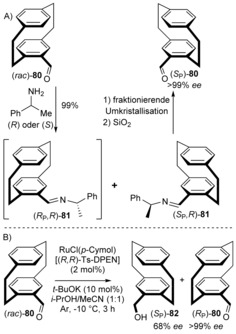
Access to enantiopure 4‐formyl[2.2]paracyclophane (**80**). (*R*,*R*)‐Ts‐DPEN=https://www.chemicalbook.com/ChemicalProductProperty_EN_CB1180813.htme.

Racemic 4‐formyl[2.2]paracyclophane (**80**) can be easily enantioenriched by fractional crystallization of the diastereomeric mixture of the Schiff base derivative **81** with (*R*)‐α‐phenylethylamine. The enantiomerically and diastereomerically pure imine is easily hydrolyzed under SiO_2_/acidic conditions to afford (*S*_P_)‐aldehyde **80** (Scheme 8). The enantiomeric excess can be conveniently monitored by ^1^H NMR spectroscopy of the imine hydrogen atom. This procedure has proven efficient and convenient for an *ortho*‐hydroxyformyl PCP derivative, a key building block and chiral ligand for asymmetric catalysis.^59^ Recently, Benedetti, Micouin, and co‐workers reported an efficient kinetic resolution procedure involving asymmetric transfer hydrogenation (Scheme 8 B), which gives the desired key intermediate on a gram scale.^62^ This method can be used for the kinetic resolution and desymmetrization of difunctionalized PCP derivatives bearing an aldehyde functionality.^63^

**Scheme 9:**
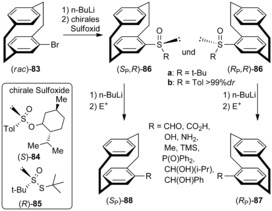
Chiral resolution of [2.2]paracyclophane sulfoxides. TMS=trimethylsilyl.

Rowlands and Seacome reported a method for the preparation of monosubstituted chiral sulfoxides **86** using readily available chiral sulfinic ester derivatives, such as toluenesulfinate **84** 64 and thiosulfinate **85**,^65^ as suitable derivatizing agents for the PCP core (Scheme 9). In contrast to the unstable Schiff bases, the diastereomeric sulfoxides **86** can be separated by column chromatography on a ten gram scale,^65^ with >99 % *ee* in the case of **86 b**.^64^ Thereafter, the sulfoxide group is cleaved by *n*‐BuLi to obtain an enantiopure lithiated PCP intermediate that can be quenched with a number of nucleophiles or can be derivatized to a chiral thiol. Essentially, this is a promising method to access a wide range of precursors in an enantiopure way.

A simple and efficient chiral resolution for racemic disubstituted pseudo‐*ortho* 4‐bromo‐12‐hydroxy[2.2]paracyclophane has been reported via diastereomeric esters of (1*S*)‐(−)‐camphanic acid, which is a key step in the synthesis of chiral *pseudo*‐*ortho*‐substituted hydroxy[2.2]paracyclophane‐based ligands.^66^

**Scheme 10:**
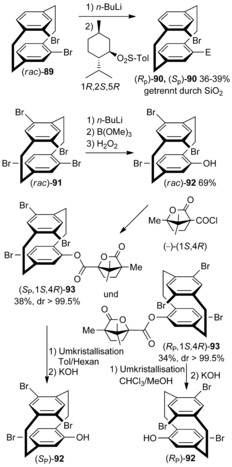
Chiral resolution of di‐ (**90**) and tetrasubstituted (**92**) [2.2]paracyclophanes.

Chujo et al. reported the chiral resolution of di‐ and tetrasubstituted [2.2]paracyclophanes (Scheme 10). Racemic pseudo‐*ortho*‐dibromo[2.2]paracyclophane *rac*‐**89** was monolithiated followed by a reaction with (1*R*,2*S*,5*R*)‐(−)‐menthyl‐*p*‐toluenesulfinate to obtain the *R*_p_,*S* and *S*_p_,*S* diastereomers, which were separated by chromatography in 39 % yield. The isolated diastereomers were reacted with *t*‐BuLi, followed by trapping with an electrophile, thereby resulting in the corresponding planar chiral molecules (*R*_p_)‐**90** and (*S*_p_)‐**90**.^67^ In a similar way, treating the chiral tetrabromo‐PCP derivative **91** with *n*‐BuLi/B(OMe)_3_ affords the racemic tribromo alcohol **92**. The tribromo alcohol **92** is condensed with (−)‐(1*S*,4*R*)‐camphanoyl chloride to give a diastereomeric mixture of camphanoyl esters.^68^ These camphanoyl esters (*S*_p_,*S*,*R*)‐ and (*R*_p_,*S*,*R*)‐**93** are purified by fractional recrystallization to yield each diastereomer in over 99 % *dr*. Upon purification, the esters are cleaved and the PCP skeleton is converted into the chiral tetrasubstituted [2.2]paracyclophane **92**.

## 4 [2.2]Paracyclophanes as Modular Building Blocks: Application‐Based Design Considerations of π‐Stacked Conjugated Polymers, Macrocycles, and Devices

Transition‐metal‐catalyzed reactions for the formation of carbon–carbon bonds have become an essential tool in synthetic organic chemistry. In combination with different carbon‐based nucleophiles, for example, aryl derivatives of magnesium (Kumada–Corriu), tin (Stille–Migita),^69^ boron (Suzuki–Miyaura),^70^ zinc (Negishi),^71^ and silicon (Hiyama)^72^ have revolutionized the chemical and life science industries.^73^ These versatile procedures allow the large‐scale synthesis of substances as diverse as pharmaceuticals, agrochemicals, and advanced electronic materials.^74^ Related cross‐coupling reactions, such as Sonogashira—Hagihara to couple terminal alkynes^75^ or Mizoroki–Heck to couple alkenes,^76^ have worked wonders in the development and progress of chemical synthesis.

### 4.1 Functionalized [2.2]Paracyclophanes as Modular Building Blocks in π‐Stacked Conjugated Polymers

In the last couple of years, milder, broader, and more efficient transition‐metal catalysts have dramatically changed the face of modern paracyclophane chemistry, and a new dimension has been opened for the exploration of the PCP scaffold towards novel π‐stacked conjugated polymers.

**Figure 6:**
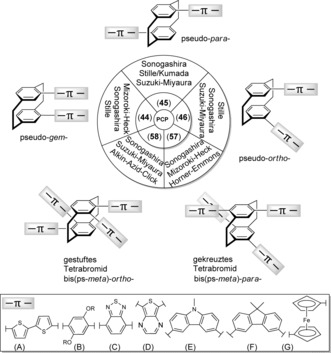
Overview of π‐stacked conjugated polymers with [2.2]paracyclophane scaffolds synthesized by metal‐catalyzed cross‐coupling reactions.

[2.2]Paracyclophanes containing halides and pseudohalides represent versatile modular molecular building blocks in the design and development of hole‐transporting materials^77^ and helically structured chiral macrocycles.^78^ These building blocks can be incorporated into numerous π‐stacked conjugated polymers to introduce the innate physical/chemical properties of PCP such as their planar chirality and layered structure.^79^ As Figure 6 illustrates, the Suzuki–Miyaura, Sonogashira–Hagihara, and Mizoroki–Heck reactions are the most common Pd‐catalyzed coupling routes for the assembly of π‐stacked conjugated polymers containing iodo‐, bromo‐, vinyl‐, ethynyl‐, and formyl‐substituted [2.2]paracyclophanes as key components in their skeleton.^80^ Structurally different PCP derivatives such as pseudo‐*geminal*‐ (**44**), pseudo‐*para*‐ (**45**), and pseudo‐*ortho*‐dibromo‐[2.2]paracyclophane (**46**) allow the construction of various π‐stacked conformations such as linear, zig‐zag, and fully stacked structures. Different π‐systems, such as donor (fluorene) and acceptor (2,1,3‐benzothiadiazole) segments can be alternately incorporated as co‐monomers to tune the energy levels and charge‐transfer properties in the resulting π‐stacked polymer system.^29^ In a similar way, charge‐transfer polymers consisting of [2.2]paracyclophane‐based dithiophenes, carbazoles, thieno[3,4‐*b*]pyrazine, and ferrocenyls (Figure 6, bottom) in the main chain have been prepared using palladium‐catalyzed synthetic procedures.^79^ The electronic and optical properties of the polymer backbone can also be altered through the external co‐monomers (Figure 6 A–G).

Hopf and co‐workers have reported a series of diverse ethynyl[2.2]paracyclophanes obtained through a Pd‐catalyzed Sonogashira–Hagihara cross‐coupling reaction of their corresponding brominated and/or formylated precursors.^81^ These carbon‐rich acetylene‐tagged cyclophanes can be employed as new building blocks in copper‐catalyzed alkyne‐azide click (CuAAC) reactions as well as multifold Sonogashira–Hagihara cross‐coupling reactions to design and build complex extended molecular scaffolds.

### 4.2 Chiral Di‐ and Tetrasubstituted [2.2]Paracyclophanes: Recent Advances in Chirality, Helicity, and Macrocyclization

**Figure 7:**
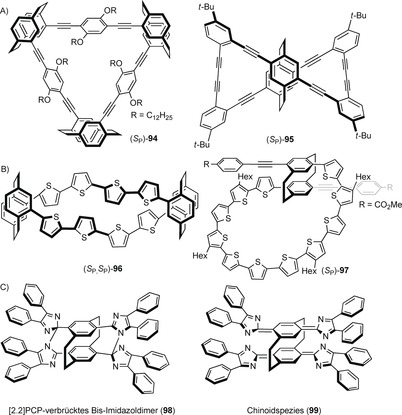
Chiral di‐ and tetrasubstituted [2.2]paracyclophane‐based helical macrocycles and PCP‐bridged bisimidazole dimer (quinoid species).

Based on a disubstituted planar‐chiral pseudo‐*ortho*‐diethynyl[2.2]paracyclophane and tetra‐ethynyl[2.2]paracyclophane obtained from **90** and **92**, Chujo, Morisaki, and co‐workers have intensively studied the enhancement and control of the circularly polarized luminescence (CPL) of the optically active π‐conjugated oligo(*p*‐phenylene ethynylene).^82^ A series of optically active cyclic conjugated structures, for example, **94** and propeller‐shaped structure **95**, as depicted in Figure 7 A, have been created by the Pd‐catalyzed Sonogashira–Hagihara procedure on [2.2]paracyclophane cores with *para*‐phenylene‐ethynylene moieties.^83^ Recently, various helical macrocyclic oligothiophenes with stereogenic [2.2]paracyclophane scaffolds having pseudo‐*ortho* and pseudo‐*para* orientations have been reported using planar‐chiral pseudo‐*ortho*‐PCP moiety **96** and pseudo‐*para*‐PCP moiety **97**, bridged by oligothiophene chains (Figure 7 B).^84^ Multiple regioselective halogenation and cross‐coupling reactions have been used to access these macrocycles.^85^ It was observed that the [2.2]paracyclophane core introduces a three‐dimensional perturbation into a nearly planar macrocyclic oligothiophene accompanied by macrocyclic helical chirality.

Abe and co‐workers have investigated [2.2]paracyclophane‐bridged bis‐imidazole dimer **98** to investigate photosynergetic effects and their potential application as multiphoton‐gated optical materials.^86^ The [2.2]paracyclophane‐bridged bis‐imidazole dimer is composed of two photochromic units and is an ideal scaffold to study stepwise two‐photon‐gated photochemical phenomena (Figure 7 C). The two imidazole rings are constrained and restrict the diffusion of the radical, hence the rate of the thermal back reactions can be tuned on time scales from sub‐microseconds to hundreds of milliseconds.^87^ Upon absorption of the first photon by **98**, a short‐living biradical species is generated and a second photon absorption results in a tetraradical species which undergoes a rapid reaction to the long‐lived quinoid **99**.

### 4.3 Chemical Vapor Deposition to Functional Surface Coatings

**Figure 8:**
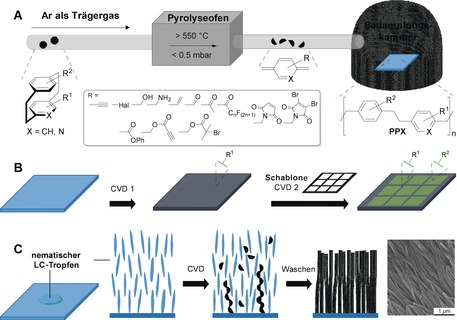
A) General procedure for the chemical vapor deposition (CVD) of functional [2.2]paracyclophanes and [2.2]heterophanes. B) Micropatterning by CVD. C) Templated synthesis of nanofiber arrays by CVD within anisotropic liquid‐crystalline droplets; SEM image reproduced from Ref. 93 with permission. Copyright 2018 *Science*, American Association for the Advancement of Science (AAAS).

[2.2]Paracyclophanes and their functionalized derivatives are well‐established precursors for the formation of poly(*para*‐xylylene) polymers (parylenes). As first described by Gorham,^88^ [2.2]paracyclophane can be cracked homolytically at the ethylene bridges at high temperatures, which generates 1,4‐quinodimethanes (*para*‐xylylene; Figure 8 A). After deposition of these reactive intermediates from the gas phase, a substrate‐independent polymerization occurs at the interface.

This process is named chemical vapor deposition (CVD). Although the strain and chirality of the PCP is lost, one of the advantages of parylene coatings generated from (functional) PCP monomers is the absence of any side products. Therefore, this CVD process has found ample application in the coating of interesting biological and optoelectronic devices.^89^ As numerous functional groups are stable under the furnace conditions, the transfer of functional groups from the [2.2]paracyclophane monomer to an interface is possible. To date, compelling results have been obtained with functionalized CVD coatings, especially with regard to three‐dimensional polymer nanostructures and bio‐interface engineering.^90^ As functionalized PCP derivatives generate reactive parylene coatings with active tunable functional groups at the interface, a generic surface‐engineering procedure becomes available. Furthermore, microstructuring by sequential CVD with patterning and nanolithography techniques has been developed (Figure 8 B).^91^ The surface‐deposited functional groups are readily accessible for post‐deposition surface functionalization, for example, by orthogonal “click” reactions of terminal alkynes with biomolecules to generate devices on a nano‐ to micrometer scale.^92^ Recently, CVD was reported within liquid‐crystal (LC) droplets. An intriguing shape control of the resulting nanofibers was observed to be dependent on the anisotropy of the liquid crystals (Figure 8 C). It is believed that the functional 1,4‐quinodimethane biradicals diffuse within the liquid crystals and follow the lattice structure of the respective LC template. For example, cholesteric, porous polymeric structures were reported when appropriate LC templates were used.^93^ This finding opens a new platform for functional polymer nanostructures, as chirality can be templated to well‐ordered 3D soft‐matter architectures and various functional groups can be introduced for post‐functionalization to accomplish sensing, filtration, or catalytic applications.

**Scheme 11:**
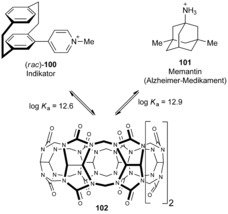
Supramolecular indicator displacement assay based on PCP and cucurbit[8]uril to spectroscopically determine the concentration of the Alzheimer's drug memantine in blood serum at physiologically relevant concentrations.

Very recently, Biedermann et al. explored the use of PCP from a structural point of view in supramolecular host–guest chemistry. They demonstrated that the PCP core is an exceptionally suitable guest for cucurbit[8]uril (CB[8], **102**), with an extraordinarily high binding affinity of *K*_a_>10^12^ m ^−1^ in water.^94^ In their study, the methylated 4‐pyridyl [2.2]paracyclophane derivative **100** (synthesized by Suzuki–Miyaura cross‐coupling employing (*rac*)‐4‐bromo[2.2]paracyclophane and 4‐pyridylboronic acid as cross‐coupling partners), was used as a competing indicator for the drug memantine (**101**), which exhibits a large Stokes shift when bound in the cavity of CB[8] (Scheme 11). An indicator displacement assay was constructed, which was able to determine the concentration of this commercially available Alzheimer drug in blood serum in a physiologically relevant sub‐ to low micromolar concentration range.

## 5 Conclusion and Outlook

The [2.2]paracyclophane scaffold is celebrating its 70th birthday and has been investigated for decades because of its unusual chemical and stereochemical features as well as applications within catalysis and materials. However, it still holds many surprises. Despite the impressive progress, synthetic challenges still remain and it is still sometimes hampered by low‐yielding functionalization methods. Significant advances in metal‐catalyzed C−H bond functionalization have been made, but the direct functionalization of [2.2]paracyclophane has scarcely been studied. New methods of C−H functionalization at the PCP backbone with excellent selectivity and improved reactivity to install a broad range of functionalities are very much desired goals, but are yet to be discovered. Investigation of the PCP derivatives as chiral ligands in asymmetric catalysis has been one of the most active areas so far; however, this scaffold with its key features of a rigid, chiral, and stable building block is emerging in other fields of research as it possesses manifold applications that are to be explored. Among them are, for example, use as a chiral drug derivative, a through‐space light/energy‐harvesting material, a CVD coating precursor, in supramolecular host–guest assays, and most noteworthy as a building block in materials with a sophisticated three‐dimensional architecture. Selective functionalization at specific positions of the PCP backbone, which allows for the incorporation of a vast range of substitutents, is particularly important from a synthetic point of view and with regard to materials perspectives. Designing chiral functional PCPs for CVD may open a new dimension in the development of helically twisted nanofibers and thin films. PCPs as functional molecules are now evolving toward functional materials of significant topological complexity.

All this fascinating research is continuously bottle‐necked by the challenging chemistry of the [2.2]paracyclophane, which demands for convergence between synthesis and engineering. Despite remaining challenges, however, it can be anticipated that focusing on [2.2]paracyclophanes will stimulate further research and will be scientifically rewarding in countless ways. We are looking forward to exciting new applications being uncovered in the near future.

## Conflict of interest

The authors declare no conflict of interest.

## Biographical Information

*From left to right: S. Bräse*, *Z. Hassan, D. Knoll, and E. Spuling Zahid Hassan studied chemistry at HEJ Research Institute of Chemistry (V. U Ahmed, 2005), at Leibniz University of Hannover (H. Duddeck, 2008), and received his PhD at Institute of Chemistry, Leibniz‐Institute for Catalysis e. V. an der University of Rostock (P. Langer, 2012), studying mechanistic and synthetic applications of metal‐catalyzed coupling reactions. After an IBS Fellowship at the Centre for Self‐assembly and Complexity, POSTECH (K. Kim, 2014), he accepted a faculty position at the University of Nizwa. Since 2017, he has been an associate at the Institute of Organic Chemistry (with S. Bräse), at the Karlsruhe Institute of Technology (KIT). His research centers on applying metal‐mediated catalysis in synthesis and design of synthetic materials. Eduard Spuling obtained his BSc in Chemistry at the Karlsruhe Institute of Technology (KIT) in 2013. After an internship working on OFETs for BASF in Singapore in 2014, he finished his MSc in Chemistry in 2015, and PhD thesis in 2019 under the mentorship of S. Bräse, working on organic light‐emitting materials based on the [2.2]paracyclophane scaffold. Currently he is a postdoctoral research fellow at the University of St Andrews working on organic electronics with E. Zysman‐Colman. Daniel M. Knoll obtained his BSc in Chemistry at the Karlsruhe Institute of Technology (KIT) in 2014. He finished his MSc in Chemistry in 2016 and currently is a PhD candidate in the group of S. Bräse, working on heteromultimetallic photoredox catalysts featuring the [2.2]paracyclophane scaffold. Stefan Bräse studied in Göttingen, Bangor (UK), and Marseille and received his PhD in 1995 with A. de Meijere in Göttingen. After postdoctoral research at Uppsala University (J. E. Bäckvall) and The Scripps Research Institute (K. C. Nicolaou), he began his independent research career at the RWTH Aachen in 1997. In 2001, he finished his Habilitation and moved to the University of Bonn as Professor for organic chemistry. Since 2003, he has been Professor at the Karlsruhe Institute of Technology (KIT) and since 2012 also Director of the Institute of Toxicology and Genetics (ITG) at the KIT*.